# Minor alleles are associated with white rust (*Albugo occidentalis*) susceptibility in spinach (*Spinacia oleracea*)

**DOI:** 10.1038/s41438-019-0214-7

**Published:** 2019-12-01

**Authors:** Henry O. Awika, Thiago G. Marconi, Renesh Bedre, Kranthi K. Mandadi, Carlos A. Avila

**Affiliations:** 1 Texas A&M AgriLife Research and Extension Center, Weslaco, TX 78596 USA; 2 Department of Plant Pathology and Microbiology, College Station, TX 77843 USA; 3 Department of Horticultural Sciences, Texas A&M University, College Station, TX 77843 USA

**Keywords:** Plant breeding, Genetic markers

## Abstract

Minor alleles (MA) have been associated with disease incidence in human studies, enabling the identification of diagnostic risk factors for various diseases. However, allelic mapping has rarely been performed in plant systems. The goal of this study was to determine whether a difference in MA prevalence is a strong enough risk factor to indicate a likely significant difference in disease resistance against white rust (WR; *Albugo occidentalis*) in spinach (*Spinacia oleracea*). We used WR disease severity ratings (WR-DSRs) in a diversity panel of 267 spinach accessions to define resistant- and susceptibility-associated groups within the distribution scores and then tested the single-nucleotide polymorphism (SNP) variants to interrogate the MA prevalence in the most susceptible (MS) vs. most resistant (MR) individuals using permutation-based allelic association tests. A total of 448 minor alleles associated with WR severity were identified in the comparison between the 25% MS and the 25% MR accessions, while the MA were generally similar between the two halves of the interquartile range. The minor alleles in the MS group were distributed across all six chromosomes and made up ~71% of the markers that were also strongly associated with WR in parallel performed genome-wide association study. These results indicate that susceptibility may be highly determined by the disproportionate overrepresentation of minor alleles, which could be used to select for resistant plants. Furthermore, by focusing on the distribution tails, allelic mapping could be used to identify plant markers associated with quantitative traits on the most informative segments of the phenotypic distribution.

## Introduction

Genetic association studies can be used to determine whether a genetic variant is associated with a particular trait. If an association is present, a particular allele, genotype, or haplotype will be seen more often than is expected by chance in individuals or groups carrying the corresponding trait^1^. Likewise, an individual carrying one or two copies of a particular allele is more likely to present the associated trait, such as susceptibility to a disease. The identification of the groups or individuals at high risk of developing a particular disease can be a daunting task, given that many major alleles may also be risk factors^2^. Shifts in allele frequencies are also common^3,4^ and can be challenging to track in fast-paced breeding programs relying on genome-wide association (GWA) mapping strategies alone. Therefore, the aim of this study was to determine whether particular minor alleles (MA) could be found more often than by chance in relation to a simple distribution using white rust disease severity ratings (WR-DSRs), in a diverse collection of spinach (*Spinacia oleracea*) accessions as a test model.

WR in spinach is caused by *Albugo occidentalis*, an oomycete obligate pathogen that attacks the vegetative and flowering structures of the affected plants and causes yellow lesions on the upper leaf surface and white pustules on the abaxial side, resulting in severe yield losses^5^. Resistance to WR in spinach is reported to be polygenic^5,6^; however, the mapping of its genic features is far from conclusive. It is perhaps not surprising that the genetic and molecular basis of resistance to WR is not well understood in spinach, and new approaches are required to elucidate the genetic and molecular basis of this resistance. One possibility is to perform a GWA to target minor alleles implicated as risk factors in susceptibility to the disease^7^. Determining the distribution of such alleles in diseased plants might illuminate the underpinnings of minor alleles important for WR susceptibility in spinach.

Compared to human studies, it is relatively easy to control and monitor the population structures and family mating systems of cultivated crops, the no sedentary nature of humans, coupled with ethical issues, means that providing the same treatment or exposure to disease is not a trivial matter^8,9^. Despite these challenges, association studies comparing the contribution of alleles in different disease outcomes are more common in humans and animals than in plants, with the number of published articles (and the value of investments) exploring human allelic associations to diseases significantly dwarfing the numbers in plants to date^8,10^. It is common to partition human study subjects into retrospective and prospective cohorts, and case and control groups^11^. Retrospective cohort studies are usually conducted on data that already exist, and the disease exposures are defined before the existing outcome data are explored to determine whether exposure to a risk factor is associated with a statistically significant difference in the disease development rate^11,12^. These studies then follow the participants for a defined period to assess the proportion that develop the outcome/disease of interest^10^. After this, the genetic associations can be explored to determine whether single-locus alleles or genotype frequencies (or more generally, multi-locus haplotype frequencies) differ between two groups of individuals, such as diseased subjects and healthy controls^11^. Studies implementing such experimental designs have led to successful identification of loci harbouring risk alleles for important complex diseases^9,10,13^. Just as GWA studies (GWASs) were adapted from human studies and became even more successful in plants^8,13^, we propose that it is possible to adapt a related methodology involving the partitioning of study populations for use in genomic studies of plants.

Unrelated, random individuals are included in retrospective cohort (segmented) studies of human diseases; therefore, we used individuals from plant populations that were assumed to be unrelated and random. We determined our study segments based on the WR-DSRs that we phenotyped. Studies of segmented populations can assess a range of outcomes, allowing an exposure level to be rigorously assessed for its impact on developing disease^11^. Our segment definition drew on the strength of using a random, nonbiased selection of individuals, since the underlying genetic interventions in the defined segments were naturally occurring. The intention was to determine whether the difference in allelic prevalence is a strong enough risk factor to predict WR disease outcomes. By comparing these results with those of a GWAS in which relatedness among individuals is accounted for, we highlight the potential use of this technique in predicting breeding markers associated with WR in spinach.

## Materials and methods

Two parallel analyses were conducted: (1) a permutation-based basic allelic association to determine the relationship between the minor allele distributions and the WR-DSR, and (2) a GWAS adjusting for population and kinship structure to compare with the markers identified in the basic allelic association mapping.

### Plant materials, growth environment, disease inoculation, and phenotyping

A panel of 267 accessions described in our previous report^14^ was used in this study. Briefly, the 267 spinach accessions consisted of a diverse panel with accessions from 33 countries and part of a collection maintained and provided by the USDA-National Plant Germplasm System (NPGS) at Ames, Iowa, USA. A detailed description of each accession can be found at https://npgsweb.ars-grin.gov/gringlobal/search.aspx. The 267 accessions were grown at the Texas A&M AgriLife Research and Extension Center located at Weslaco, Texas, USA, at a latitude of 26° 9′ 30′′N and a longitude of 97° 57′43′′W. The plants were grown in a randomized complete block design with three replicates per accession. Each plot contained two rows of ~14 plants spaced at ~10-cm intervals, with 15 cm between the two rows. Each plot was 1.22 m from the next adjacent plot. Conventional agronomic practices for spinach were used, from land preparation to the last date of data collection. The crop was fertilized with a generalized N–P_2_O_5_–K_2_O rate of 135–84–90 kg/ha.

The natural field inoculation was accomplished by planting crops in previously infected field. The experimental plots were intercalated with rows of the susceptible control cultivar “Monstrueux de Viroflay” to uniformly spread the disease. WR dispersal and infection is favoured by cool and humid conditions with mild wind and water conditions^5,15^; therefore, the spinach field was irrigated as needed to improve the spatter spread of the spores and enhance humidity within the plant canopy. The WR symptoms were evaluated on a per-plot basis, as described by Dainello et al.^16^. Briefly, the WR-DSR scores were based on the surface area covered by WR lesions as a percentage of total surface area of a plot. The percent ratings were categorized in 1%, 5%, and then at intervals of 5% up to 50%.

### Tissue collection, DNA isolation, genotyping, and SNP calling

Tissue handling, DNA isolation and library preparations, and genotyping and single-nucleotide polymorphism (SNP) calling were performed as previously described by Awika et al.^14^, and the genotyping framework followed the double digest restriction site-associated DNA (ddRAD-seq) genotyping-by-sequencing protocol described by Peterson et al.^17^. The Illumina short-read sequencing (HiSeq 2500) and demultiplexing using individual indexes were performed using the Texas A&M AgriLife Genomics and Bioinformatics services. The paired-end raw sequencing reads (150 bp) were subjected to filtering to obtain high-quality reads for the downstream analysis. The raw reads were filtered and trimmed to remove adapter contamination and low-quality (average quality score ≤ 20) or ambiguous sequences with uncalled nucleotides (<5% uncalled bases). The filtering and trimming of the raw reads were performed using an in-house pipeline developed using Python^18^ (https://github.com/reneshbedre/RseqFilt). The high-quality cleaned sequence data were aligned to the draft spinach reference genome (v1)^19^ using the bowtie2 alignment tool^20^.

The 267 spinach accessions were genotyped using Stacks (v1.48)^21,22^, based on the draft spinach reference genome (v1). In brief, various modules of the Stack pipeline (pstacks, cstacks, sstacks, and rxstacks) were used to identify and filter the genotypes^21^. The Ada cluster of the TAMU High Performance Research Computing (http://hprc.tamu.edu/) was used to perform the bioinformatics analysis. The SNP pipeline-end cleanup criteria also included the removal of SNPs not anchored onto the six published draft chromosomes^19^. A final 6166 SNPs in VCF v4.2^23^ were available for the downstream analyses of which 6,111 were used in this study. The SNPs in the spinach accession data can be downloaded from the supplementary table presented by Awika et al.^14^.

### Adjusting for population and interallelic stratification

STRUCTURE (v2.3.4)^24^ and a kinship matrix^25^ were used to adjust for population stratification and allele sharing^26^ before running the GWAS (see below). STRUCTURE uses identity-by-state similarity, assuming that two random alleles drawn from the same locus are the same. An admixture model was run, with the distance of an individual from itself set to 0. The protocols for using STRUCTURE, visualization in STRUCTURE HARVESTER^27^, the associated algorithms^28,29^, and the kinship matrix (*K*) were described previously by Awika et al.^14^, except that, in this study, 5000 burn-ins instead of 1000 burn-ins were used in STRUCTURE, and 17 rather than 15 replications were performed for each of the seven population assumed (*K*). This was to further improve the accuracy of convergence toward reliable estimate of allele frequency in each (hitherto arbitrary) subpopulation.

### Distribution and accuracy of phenotypic prediction

A modified form of the retrospective cohort of human diseases^30^ and the statistical principles of case–control genotypic and allelic associations^31–33^ were used to define the study samples. The study groups (segments) were retrospectively defined based on the distribution of the WR-DSR scores, providing a random, nonbiased inclusion of individuals in each segment. The best linear unbiased predictor estimates (BLUPs), least square means (LSM), and arithmetic means (ArithMean) of the WR-DSR scores were used to construct the distribution.

The BLUPs and LSMs were determined by running the restricted maximum likelihood (REML) model on the three replicate WR-DRS scores in JMP Statistical Software v14 (SAS Institute, Cary, NC, USA) using the following formula: Y*ij* = µ + U_*i*_ + W_*ij*_ + *e*, where *µ* is the mean WR-DSR for the whole population, *U* is the random plot mean, *W* is the random deviation of the *j*th replicate from the *i*th plot mean, and *e* is the residual error accounting for plot-to-plot differences. To test the accuracy of the BLUPs for predicting the true phenotypic means, the correlations between the LSMs and BLUPs and between the ArithMean and BLUPs were determined. One advantage of the BLUP estimates is that they have a reduced mean square error within the linear plot estimators and thus deviated less from the mean of the realized plot phenotypic values^34,35^. The data were then split into a random test sample (25%) and a training set (75%) to test the model fitness in different sample sizes. The slopes, *R*^2^, Akaike’s information criteria (AIC), and Bayesian information criteria (BIC) were compared for the three equations (the whole sample, the 25% sample and the 75% sample).

To partition the data into segments consisting of individuals with high WR-DSRs (i.e., most susceptible [MS]) and individuals with low WR-DSRs (i.e., most resistant [MR]), the whole sample (the “population” of 267 individuals) was also tested for the sample distribution statistics of normality, skewness, and kurtosis. For these measures, a “sample statistics” computation was used because the material in this study is part of a larger diversity population maintained by the USDA. In the strict context of this study, the term “population” is used to refer to the 267 accessions used. The D’Agostino-Pearson Omnibus test^36,37^ was applied at *α* = 0.05 to assess normality, while skewness (S), a measure of asymmetry^38^, was determined as S(G_1_) = [√*n*(*n* − 1)/(*n* − 2)]·g_1_^38^. The standard error of skewness (SES) was calculated as SES = √[6*n*(*n* − 1)/(*n* − 2)(*n* + 1)(*n* + 3)]^38^, where *n* is the sample size and g_1_ is the squared variance (*σ*^4^).

To assess the deviations in the distribution variance, kurtosis was determined to measure whether the variance results from infrequent extreme deviations or frequent modestly sized deviations^39^. This established how reasonable the distributions of the intermediate values and the extreme values are for the two WR-DSR distribution tails^40^. Kurtosis (G_2_) was determined as G_2_ = (*n* − 1)/(*n* − 2)(*n* − 3).[(*n* *+* 1)g_2_ + 6]^38^, and the standard error of kurtosis (SEK) was calculated as SEK = 2(SES)√[(*n*^2^ − 1)/(*n* − 3)(*n* *+* 5)]^41^, where *n* is the sample size and g_2_ is the squared variance (*σ*^4^).

### Phenotypic clustering (*k*)

The data, showing satisfactory (nonsignificant) kurtosis and skewness, was partitioned into four quartiles or segments^42^, Q1–Q4, each containing ~66 plots (accessions) used in the allelic tests. To define the quartile intervals, the nearest-rank method was applied^42,43^: *n* = [(*P*/100)*N], where *n* is the ordinal rank, *P* is the percentile in question, and *N* is the total number of ranks in the ordered list. For the cross-validation of the segments, the entire population was divided into mutually exclusive five percentile segments (*k* = 10). Each *k* consisted of ~13 accessions with WR-DSR scores, and ~10 accessions were drawn from each of the *k* segments for the downstream allelic analysis. The phenotypic raw data and assigned clustering can be found in Supplemental Tables ST1 and ST2.

### Permutation within clusters and testing the hypothesis of allelic associations

The frequencies of alleles in the two opposite segments in the WR-DSR distribution were compared, i.e., the segments containing individuals with WR-DSR scores lower or higher than the mean population. The pairs of segments consisted of ~20 individuals (for the 10 *k*, five-percentile segments) and ~130 individuals for the Q1 vs. Q4 and the Q3 vs. Q4 tests. BLUPs and genotypic data were used to perform the basic allelic permutation tests to provide empirical *P* values that also control the familywise error rate^31^. In computing the allelic test for each segment pair, the individual members of segments with a mean larger than the population mean were populated with a phenotype code of 1. The individual members of the corresponding segments with a mean smaller than the population mean were populated with the code 2. The plots not included in either pair were coded as 0 (missing)^31,44^.

The tested hypothesis was that the allelic coefficients associated with WR = 0, while the alternative hypothesis was that these allelic coefficients ≠ 0 (i.e., a relationship exists between the specific allele and the WR). The max(T) permutation method was applied in PLINK and gPLINK (http://zzz.bwh.harvard.edu/plink/index.shtml; http://zzz.bwh.harvard.edu/plink/gplink.shtml)^45^, with 10,000 iterations for all SNPs. Max(T) performs all specified permutations without dropping what might be nonsignificant alleles^45^. The benefit of this is that two sets of empirical significance values could then be calculated: the pointwise estimates of the significance of an individual SNP and a value that controls for the fact that thousands of other SNPs were also tested. This was achieved by comparing each observed test statistic against the maximum of all permuted statistics (over all SNPs) for each segment pair.

The permutation-based Max(T) test uses a hypergeometric distribution to compute probabilities and thus does not depend on any large-sample distribution assumptions^45,46^. The hypergeometric distribution was especially important in this study because it allows for the classification of data into two mutually exclusive samples with random draws within the sets of samples^47,48^. The probability tests from these permutations could generate exact (comparable to Fisher’s exact) empirical significance values^48,49^ that are valid in small samples such as the segments used here where the likelihood ratio and Pearson tests become less reliable^46,50^. Because the permutation schemes preserve the correlational structure between SNPs, these tests provided a less stringent correction for multiple testing than the Bonferroni correction (BC), which assumes all tests are independent. In fact, because the corrected *P* value was of interest, it was not necessary to demonstrate genome-wide significance levels beyond 0.05 or 0.01^45^. A *P* value ≤ 0.01 was used to declare a significant association for the allelic tests, and an odds ratio (OR) > 1 was used to declare risk.

The pre-analysis thresholds were minor allele frequency (MAF) = 0.01, and max SNP missingness = 0.1; i.e., to be incorporated into the analysis, a SNP must be present in at least 90% of the accessions (individuals), otherwise it was omitted. This stringency was increased from the 80% used in the GWA, which was performed in parallel to the allelic tests (described below) to penalize what was believed to be-possible spurious associations during the multiple test permutation correction^51^. Individual missingness was set at 0.1; i.e., an accession was included in the analysis only if at least 90% of the SNPs were present in it.

### Genome-wide association studies

Since basic allele tests may require corroboration using other tests^51,52^, a GWAS was conducted using all 267 accessions and 6111 SNP variants. The GWA approach was implemented in TASSEL v5.2.52 and corrected for the population structure and possible kinship (as described above). For the purpose of this report, the significant signals identified in the GWAS were used as a baseline to assess the efficiency of the allele-based method (in Q1 vs. Q4, in Q2 vs. Q3, and in the five-percentile segments). A basic genotypic association full (GAF) model was also performed for the Q1 vs. Q4 pair. The difference between the GAF and the allelic association tests is that instead of coding individuals as 1 or 2, the GAF uses the actual BLUPs to compute the genotypic association tests, while using similar permutation parameters to those specified for the allelic tests. The associated graphic plots were generated using qqman v0.1.4^53^ in R, ggplot2 v3.1.0^54^ in R, DAAG v1.22.2^55^ in R, and e1071 v1.7–1 (https://www.rdocumentation.org/packages/e1071) in R, as well as Excel and jvenn^56^.

### Determining the efficiency of the allelic association

The markers identified via the GWA model were considered as the reference for true associations and were used to determine the efficiency of the allelic mapping using the case–control models at with the Q1 vs. Q4 and Q2 vs. Q3 comparisons, and the full model GAF. To achieve this, the “true” signals in the GWA model were used to standardize the ratio (*z*/*T*) of matching markers in the other methods as a factor of the GWA signals (L) and, thus:, (*z*/*T*)c ∙ Lkc, where *z* is the number of markers identified using the allelic or GAF model that were also identified in the GWAS; *T* is the total markers identified using the allelic or GAF model, and *k* is the efficiency multiple factor for model c_1_ compared to model c_n_.

### Minor allele discrimination and realignment

In order to evaluate how well SNP markers identified by allelic mapping can discriminate between WR-resistant and susceptible individuals, we compared the MAF of the MR accessions within the fifth percentile of the WR-DSR distribution tail vs. the MAF in the corresponding fifth percentile WR-DSR distribution tail consisting of the MS accessions. A disproportionately high MAF for a marker would suggest that the marker is a susceptibility factor, while a disproportionately low MAF would suggest a resistance factor. To be considered disproportionately represented, we defined that the frequency of the allelic base at a SNP locus had to be less than half the frequency of the corresponding allele on the second strand, and significantly lower than the mean of MAFs in the second strand (at *α* = 0.01).

To test the efficacy of this method, we determine if the significant markers could discriminate between WR resistance or susceptibility among the MR (WR-DSR < 20) and 10 MS (WR-DSR > 40) accessions (see data partitioning, Supplementary Table ST2). To do this, we realigned and determined the phylogeny of the nucleotide sequences of each of the 20 accessions at the significant SNP loci using MEGA X^57^ unweighted pair group method with arithmetic mean^58^. Phylogeny was tested using 500 bootstrap replications^59^ for the combined 20 (10 MR and 10 MS) accessions. The substitution model based on the maximum composite likelihood method^60^ was implemented for the nucleotides at the significant polymorphic sites in each of the accession. We used the default settings of uniform rates and assumed homogenous pattern among lineages. Pairwise deletion was selected for sites with gaps or missing data.

### Heritability

Both the broad-sense heritability (*H*) and the narrow-sense heritability (*h*) were determined as previously described^14^. Briefly, *H* was computed on a line mean basis using the variance components generated in the REML model, with replicates nested in three blocks in a single location: *H* = *V*_*l*_/(*V*_*l*_ + (*V*_*l*_·*V*_*r*_[Block])/3 + Ve/3)), where *V*_*l*_ is the variance within a line, *r* is the replicate, and *e* is the residual.

Narrow-sense heritability (*h*) was calculated based on the marker mean genetic and residual variances. Since we specified the population parameters previously determined (P3D) in the optimal compression model (in TASSEL), *h* was obtained from the mean genetic variance and the mean residual variance in the compression models: *h* = (*σ*_*a*_^2^)/(*σ*_*a*_^2^ + *σ*_*e*_^2^), where *σ*_*a*_^2^ is the mean additive variance of the marker allele and *σ*_*e*_^2^ is the variance of the residual (error).

## Results

### Defining the distribution, data segmentation, and accuracy of phenotypic prediction

**Fig. 1:**
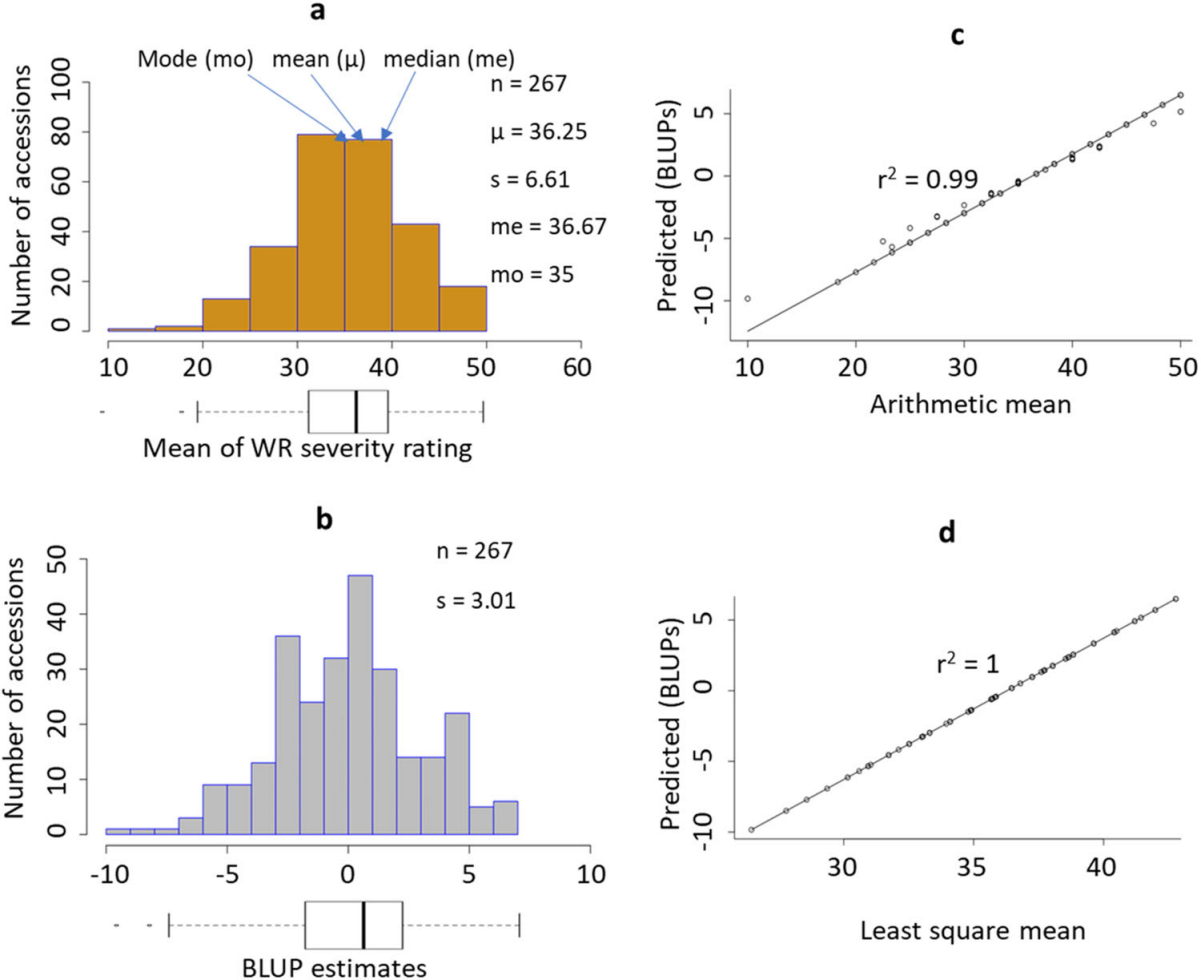
Distribution of WR-DSR and accuracy prediction test. Distributions of the arithmetic mean (**a**) and BLUP estimates (**b**) for the WR-DSR scores in the sample population are displayed. A corresponding box plot is shown below each distribution histogram. The correlations between the BLUPs and the arithmetic mean **c** and between the BLUPs and the LSM **d** are also shown. s, standard deviation of population sample.

**Fig. 2:**
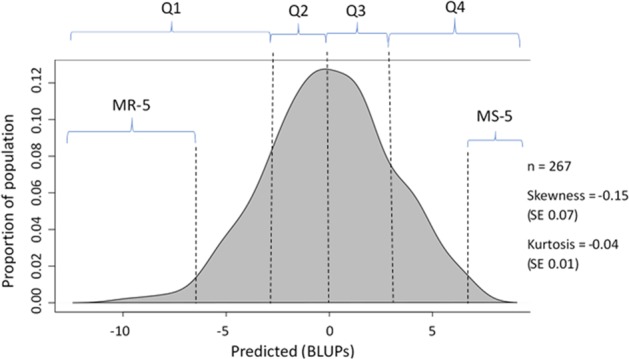
Density plot of the BLUP estimates. The segments for the most resistant 5% of samples (MR-5), the most resistant 25% of samples (Q1), the moderately resistant 25% of samples (Q2), the moderately susceptible 25% of samples (Q3), the most susceptible 25% of samples (Q4), and most susceptible 5% of samples (MS-5) are shown. Skewness or kurtosis of >±2 is not considered significant. SE standard error.

The phenotypic distribution had a slight negative (left) skew. The ArithMean was 36.25 (sd = 6.61, range: 10–50; Fig. 1a), and a few outliers identified on the lower extremes were found to cause the observed left skew. The BLUP data (Fig. 1b) had a slight, nonsignificant, negative skew of −0.15 (se = 0.07) and a excess kurtosis of −0.04 (se = 0.01), based on two-tailed tests of skewness (Z_g1_) and excess kurtosis (Z_g2_)^41^, respectively, at the 0.05 significance level. The distribution statistics for ArithMeans and BLUPs are shown in Figs. 1 and 2.

We tested the accuracy of the BLUP estimates to predict the true phenotypic means by determining the correlation between the LSMs, ArithMeans, and BLUPs. The correlation between the ArithMeans and BLUPs was 0.99 (Fig. 1c), while between the LSMs and BLUPs it was 1 (Fig. 1d). The correlations between ArithMeans and BLUP were 0.89 and 0.87 for the 75% (201-plot) test set and the 25% (66-plot) validation set, respectively (data not shown). The differences in AIC and in BIC between the three equations were negligible when comparing the whole population (267 plots) with the test set and the validation set (Table 1), indicating that we could apply the BLUP estimates across the different sample sizes.

**Table 1:** Test statistics for model fitness.

Set	Number of plots (actual)	RSME	*R*^2^	AIC	BIC
Whole	267	3.661623	0.97	5542	5566
Test	201	3.819942	0.96	5545	5571
Valid	66	4.354772	0.97	5541	5560

*RSME* root mean square error, *AIC* Akaike’s information criterion, *BIC* Bayesian information criterion

The data were segmented as shown in Fig. 2. For the *k* *=* 10 segments, only the 5% MR-5 and the 5% MS-5 on either tail are shown. Parameter counts used in association tests of the segments are shown in Table 2. In this report, we used the term “most resistant (MR-25)” to describe the accessions with WR-DSR scores in the lower 25th percentile (i.e., Q1), and “most susceptible (MS-25)” to mean the accessions with WR-DSR scores in the upper 25th percentile (i.e., Q4). We considered the accessions with WR-DSR scores between the 25th and 50th percentiles (i.e., Q2) to be moderately resistant, while the moderately susceptible were those with WR-DSR scores between the 50th and 75th percentiles (i.e., Q3).

**Table 2:** Percentile ranges and their parameters used in allelic tests.

Percentile ranges	Ind with non-missingness	Number of SNPs	Number of accessions	Genotyping rate	Number of Ind in R group	Number of Ind in S group	Genomic inflation factor	Mean *X*^2^ statistic	Number of tests corrected for in R and S
<5	15	4725	262	0.937	10	10	1.98503	1.36259	4285
5–10	20	4725	262	0.937	10	10	1.56618	1.13646	4608
10–15	20	4725	262	0.937	10	10	1.37061	1.22006	4660
15–20	20	4725	262	0.937	11	9	1.34904	1.12663	4627
20–25	19	4725	262	0.937	10	9	2.16307	1.65384	4639
25–30	20	4725	262	0.937	10	10	1.16959	1.10623	4632
30–35	20	4725	262	0.937	10	10	1.64921	1.19752	4446
35–40	20	4725	262	0.937	10	10	1.23457	1.02834	4613
40–45	21	4725	262	0.937	11	10	1.25598	1.10425	4669
45–50	20	4725	262	0.937	10	10	1.71999	1.3079	4448

*Ind* individual accessions, *R* resistant, *S* susceptible

### Number of markers associated with WR resistance identified in the allelic tests and the genotypic tests

**Fig. 3:**
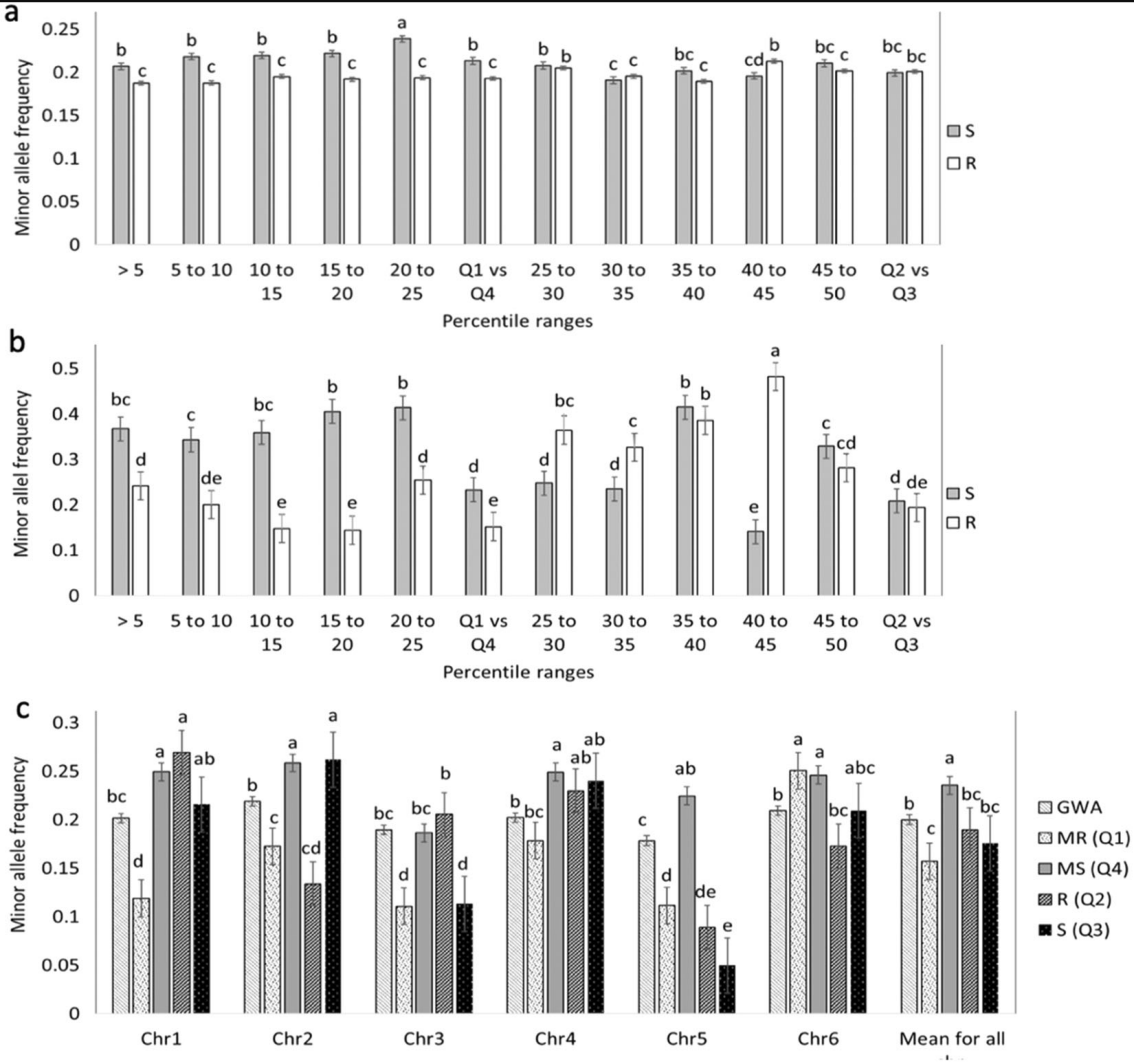
Proportion of MAFs in individuals drawn from the two tails of the BLUP distribution. Each bar represents the mean MAF proportion of ten individuals from the tail with a WR-DSR score less than the mean and greater than the mean. The ten individuals were randomly drawn from each of the percentile segments shown. **a** Mean MAF for all the 4871 SNPs. **b** Mean MAF of polymorphisms with a significant association with WR. **c** MAF per chromosome. GWA minor allele frequencies are shown against those mapped using the basic allelic association tests. Standard error bars are shown. Q quartile, MR most resistant, MS most susceptible, R resistant with reference to the mean WR-DSR, S susceptible with reference to the mean WR-DSR.

We mapped the SNPs hosting minor alleles that are associated with WR and compared the proportions of the minor alleles mapped in individuals between the corresponding population segments. The differences between the mean MAFs in the MS segments and the MR segments were significant. The MS-5 MAF was significantly higher than the mean MAF in the MR-5 group (*P* ≤ 0.008), while the MS-25 (Q4) segment had a significantly higher MAF than the mean MAF in the MR-25 (Q1) group (*P* ≤ 0.001) (Fig. 3a for all alleles and 3b for significantly associated alleles). By contrast, the intermediate quartile segments (Q2 vs. Q3) showed no significant differences except for two of the five k-segment pairs. (Fig. 3a, b). These observations indicate that a greater number of the minor alleles found to be associated with WR reside in the individuals showing the most severe WR symptoms in comparison with the resistant individuals.

### Markers identified using the allelic and GWA models

**Fig. 4:**
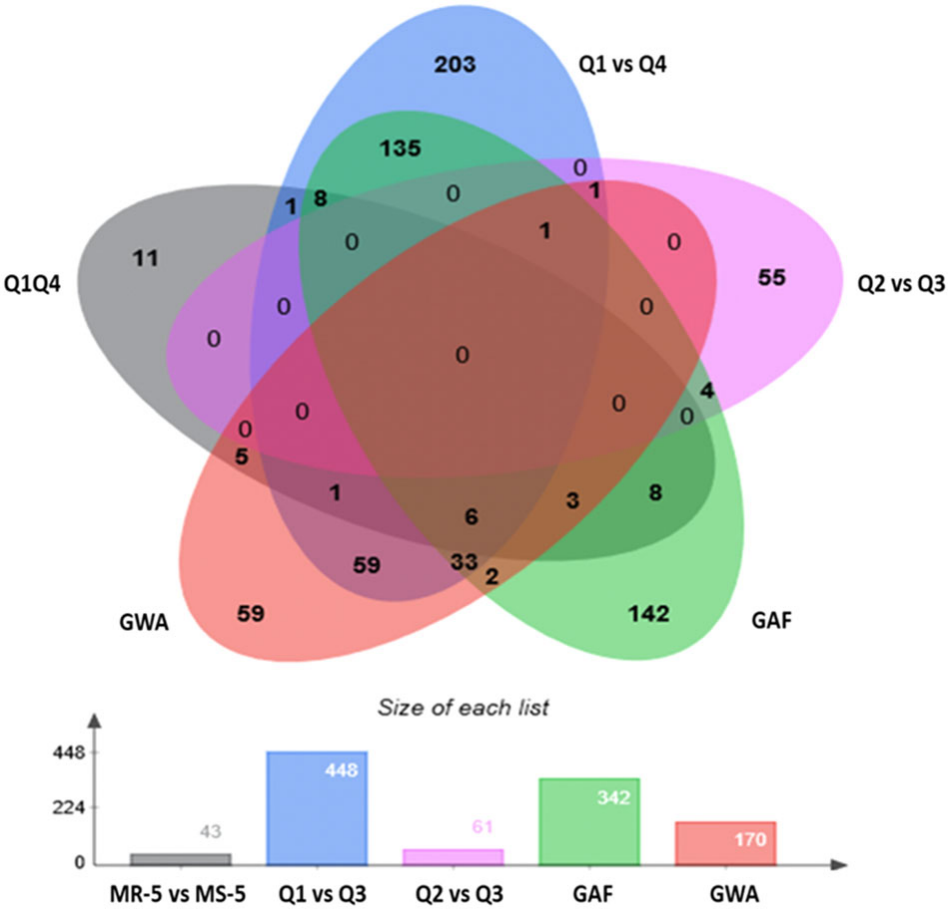
Number of markers identified in the tests (bar graph) and their similarity across the tests (Venn). Allelic tests using accessions in the most resistant and most susceptible 5% tails (MR-5 and MS-5, respectively), quartiles Q1 and Q4 (Q1 vs. Q4), and quartiles Q2 and Q3 (Q2 vs. Q3) of the WR-DSR scores across the population; basic genotypic association using a full model (GAF); and the population structure-corrected GWA analysis.

A total of 448 significant alleles (Table 3, Fig. 4) associated with WR-DSR were identified in individuals in the Q1 vs. Q4 segment comparison of ~130 individuals, while only 61 alleles were identified when the Q2 vs. Q3 segment pair was analysed (Table 3; Supplementary Tables ST3 and ST4). We compared the associated markers in the allelic tests to those in the basic full model (GAF, using the ~130 accessions in the Q1 vs. Q4 segments) and those in the GWAS model (using all 267 accessions). A total of 102 (~23%) of the 448 significant markers identified in the Q1 vs. Q4 tail comparison and only two (~3.3%) of the 61 significant markers identified using the Q2 vs. Q3 segment pair were also found to be significant in the GWA. These represented 71.8 and ~1.4%, respectively, of the 142 significant markers identified using the GWA model. The GAF identified 342 significant markers, of which 45 (~31.7%) were also found to be significant in the GWAS. The Q1 vs. Q4 and GAF analyses shared 86.7% of their significant markers (Supplementary Tables ST3, ST4, and ST6).

**Table 3:** Significant marker signals across models and associated minor allele counts compared across segments (mean standard error in parenthesis).

	GWA	GAF	Basic allelic association compared between corresponding 25% tail segments				Basic allelic association compared between corresponding interquartile segments			
Chr	#Mk	#Mk	#Mk	MR (Q1)	MS (Q4)	OR	#Mk	Q2	Q3	OR
Chr1	28	59	60	0.119 (0.03)	0.249 (0.07)	3.36 (0.41)	8	0.215 (0.07)	0.269 (0.27)	1.54 (0.42)
Chr2	26	68	95	0.173 (0.02)	0.251 (0.08)	2.83 (0.38)	15	0.133 (0.08)	0.262 (0.04)	3.52 (0.47)
Chr3	38	53	102	0.111 (0.08)	0.186 (0.04)	3.81 (0.48)	6	0.113 (0.02)	0.205 (0.05)	1.00 (0.45)
Chr4	28	78	80	0.178 (0.02)	0.249 (0.01)	3.11 (0.41)	24	0.23 (0.00)	0.240 (0.05)	1.83 (0.41)
Chr5	27	36	62	0.111 (0.09)	0.224 (0.51)	9.52 (0.39)	6	0.088 (0.02)	0.049 (0.08)	3.42 (0.82)
Chr6	23	46	49	0.250 (0.01)	0.246 (0.08)	9.88 (0.40)	2	0.209 (0.04)	0.172 (0.08)	3.76 (0.53)
^a^Number of associated alleles	na	na	na	72	376	na	na	27	34	na
% of associated alleles	na	na	na	14	86	na	na	44	56	na
Totals	170	342	448	na	na	na	61	na	na	na

*na* not-available, *#Mk* number of markers, *Chr* chromosome, *GWA* genome-wide association model, *GAF* basic genotypic association test, *Q* quartile, *OR* odds ratio

^a^Alleles in the Q1–Q4 segments

Taking the 142 markers in the GWA model as the reference for true associations, we compared the efficiency of the allelic mapping using the case–control models with the Q1 vs. Q4 and Q2 vs. Q3 comparisons, and the full model GAF. To achieve this, we used the 142 “true” signals in the GWA model to standardize the ratio (*z*/*T*) of matching markers in the other methods as a factor of the GWA signals (L). Based on comparisons of the standardized ratios, the allelic mapping in the Q1 vs. Q4 DSR tails was 7.7 times more efficient at discovering true signals than the allelic mapping using the interquartile DSR range Q2 vs. Q3, and 2.4 times more efficient than the full model GAF. These observations indicate that mapping alleles in the Q1 vs. Q4 segments better identified the loci associated with WR than mapping using the Q2 vs. Q3 segments or the GAF model in a population with a near-normal phenotypic distribution. The difference in the number of individuals and markers used, or in the method used to account for population structure and stringency in declaring test significance, might have contributed to the large difference in the markers identified using the GWA in comparison with the other models.

### MAFs determined using the GWA and allelic tests

Figure 3c presents the MAFs for each chromosome, including the MAFs from the GWA. The phenotypically less diverse Q2 vs. Q3 pair showed no significant differences in their MAFs except on chromosome 2 (chr2) and chr3. The more phenotypically diverse Q1 vs. Q4 pair showed a more dramatic difference in their MAFs, except on chr6 where there was no difference. Compared to the MAFs identified using the GWAS, the segments with higher WR-DSR scores in the allelic tests (Q2 and Q3) showed a significantly higher prevalence of minor alleles, except on chr3. The data show that the pattern of higher-risk alleles in the MS vs. MR segments was fairly well maintained across most chromosomes, except that on chr2 and chr5, the differences were more dramatic, with chr5 showing the largest difference between the Q4 MS and the rest of the segments, and between the Q4 MS and the GWA. The MAFs did not seem to correspond to the chromosomal length or chromosomal marker densities reported for this set of markers and plant population^14^. These observations suggest that the different chromosomes in spinach may contain different numbers of risk alleles for WR.

### Marker distribution

**Fig. 5:**
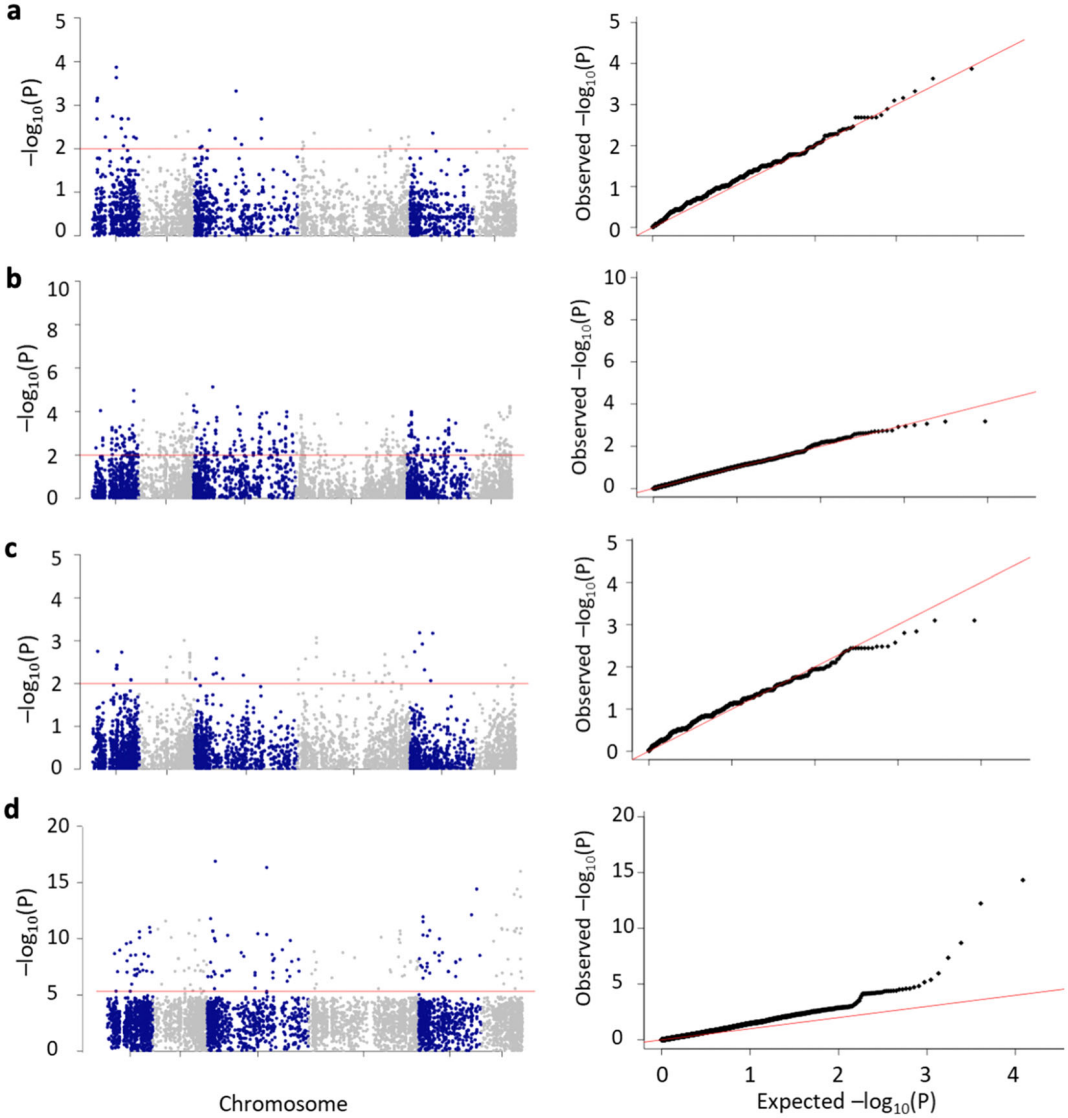
Manhattan plots of the distribution of markers across the six chromosomes. The horizontal red line is the *P* ≤ 0.01 threshold for the allelic associations (**a**–**c**) and the GWA analysis at *P* ≤ 3.06e–6 (**d**). A QQ-plot of the *P* value distribution is shown to the right of each Manhattan plot. The number of individual accessions used in the analyses were **a** 20 picked from the 5% of two extreme WR-DSR distribution tails; **b** 132 picked from quartile Q1 and Q4; and **c** 134 picked from the interquartile range, Q2 and Q3.

The plotting of associated markers in the segmented and unsegmented data (Fig. 5) revealed the presence of extended genomic regions with high-scoring association signals, which are non-chromosome specific and were identified independently of a specific association model. The population-structure-corrected GWA, which maps SNPs in linkage disequilibrium (LD), showed little difference in the pattern of the strong signals in comparison with the allele-specific mapping strategy (Fig. 4).

### Markers for WR resistance are discriminated by their extremely low minor allele frequencies in the resistant lines

In order to evaluate how well minor alleles associated with WR can discriminate between resistant and susceptible accessions for their use in molecular breeding programs, we compared the MAF in 10 MR lines within the fifth percentile WR-DSR distribution tail to the MAF in the corresponding 10 MS lines in the fifth percentile WR-DSR distribution tail. We considered that a disproportionately high MAF for a marker would suggest that the marker is a susceptibility factor, while a disproportionately low MAF would suggest a resistance factor. Based on these criteria, the majority (11) of the 15 markers identified by allelic mapping had disproportionately low MAF in the resistant plants (colored blue, Table 4), indicating their potential use as markers to select for WR resistance factors in spinach.

**Table 4:**
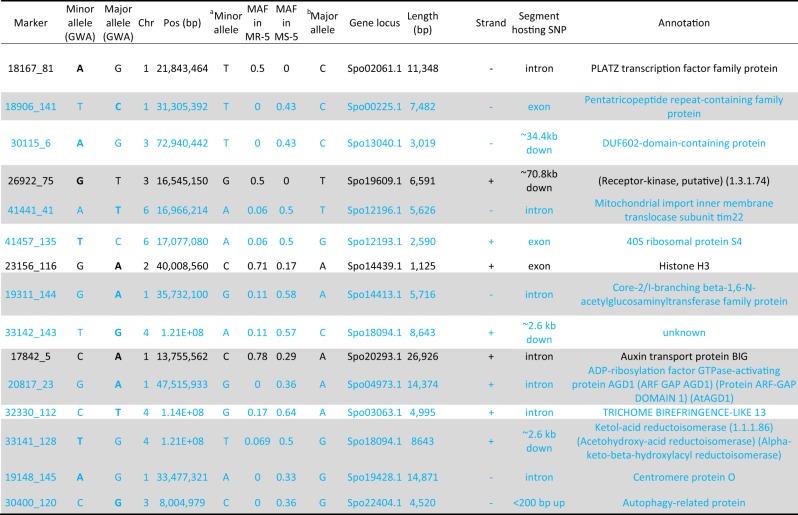
Functional annotation and allele frequency of SNP markers associated with white rust resistance identified by the allelic mapping approach at the upper and lower 5% of the phenotypic distribution.

*GWA* genome-wide association analysis, down downstream, up upstream, *MR-5* and *MS-5* most resistant and susceptible individuals in the 5% tails, respectively, *chr chromosome*. Shaded rows show the identical marker alleles in at least 200 of the 267 accessions used in the GWA and in the 20 plots found at the 5% tails of most resistant and most susceptible plots. The allele with the largest effect (in the GWA) is displayed in bold. Rows colored blue contain resistance markers associated with disproportionately low minor alleles in the white rust-resistant lines

^b^ and ^c^, Minor and major alleles in individuals in the 5% tails;

**Fig. 6:**
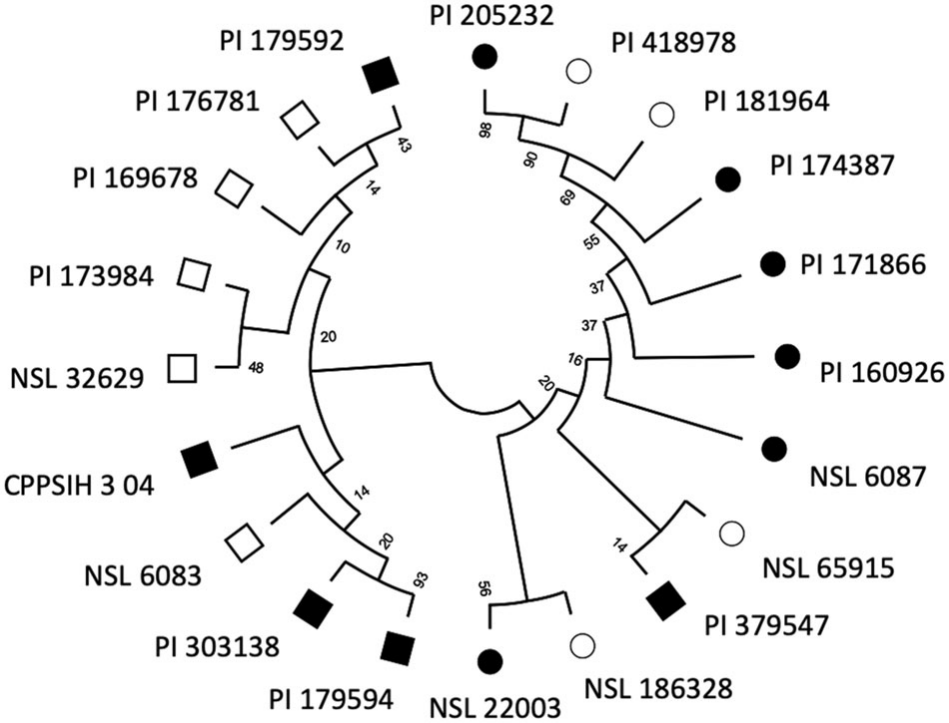
Marker-accession hereditary relationship between most resistant and most susceptible lines. Circles, susceptible lines; squares, resistant lines; filled squares and circles, significant BLUPs from REML model.

Furthermore, the realignment of the 20 sequences at the 15 significant SNP loci resulted in two divergent nodes connected to either the most resistant (MR) group on one hand and the most susceptible (MS) on the other hand (Fig. 6). The MR branch consisted of nine (PI 179592, PI 176781, PI 169678, PI 173984, NSL 32629, CPPSIH 3 04, NSL 6083, PI 303138, and PI 179594) out of ten accessions, while the MS branch had all the ten susceptible lines (PI 205232, PI 418978, PI 181964, PI 174387, PI 171866, PI 160926, NSL 6087, NSL 65915, NSL 186328, and NSL 22003). The line PI 379547, which was phenotyped as resistant was phylogenetically associated with the susceptible group and shared a node with the susceptible NSL 65915, suggesting that some of the marker signals were shared between the PI 379547 and MS accessions. These observations indicate that, with the exception of PI 379547, 9 spinach accessions phenotyped as MR may be harboring SNPs more closely associated with the 11 resistance factors (see Table 4), while the 10 MS may be mostly associated with the four susceptibility factors. The two groups can be candidate parents for developing biparental populations segregating for the WR resistance and for the resistance markers validation.

### Heritability

We observed a discrepancy in the predicted and actual heritability observed. The phenotype-based mixed model predicted 97% of the variation observed in WR-DSR, whereas the variance components explained a total of 64% of the heritability. At the marker level, the additive effect model predicted 98% of the heritable variance, even though individually, the per-marker *r*^2^ ranged between 0.00 and 0.09. These observations underpin the conundrum associated with the “missing heritability” in the GWA models.

## Discussion

### Minor alleles are more frequent in plants showing higher susceptibility to WR

The minor and major allele frequencies and identities were expected to change such that in the smaller samples, the frequency of alleles most commonly associated with disease was larger compared to that of the allele variant common throughout the entire population (equivalent to our sample of 267 accessions)^4^. Indeed, for the associated loci, the mean MAFs were significantly higher in the WR-DSR distribution tail containing the most susceptible accessions compared to the WR-DSR distribution tail containing the most resistant accessions (Fig. 3; Table 3), suggesting that these may be among the alleles most commonly associated with WR in this study. A limitation of the outlined basic allelic association methods is the possible occurrence of false positives that may be an artefact of the population structure^8^. Despite this, around five times more markers (376) of risk alleles associated with WR were identified in the most susceptible (Q4) individuals than in the most resistant (Q1) individuals (72 markers; Table 3). For the plants in the interquartile range (i.e., Q2 and Q3), however, only seven (34 vs. 27) more risk alleles were significantly associated with WR in the moderately susceptible individuals (Q3) than in the moderately resistant plants (Q2). The smaller difference in the minor allele count between Q2 and Q3 may have been due to the small phenotypic variation between these groups, as the WR-DSR is biased toward the mean^61^. This further shows that the difference in the association of risk alleles with WR is more distinct at the opposing tail distributions of the DSR.

The distribution of markers associated with WR showed no chromosome-specific pattern across the association models (Fig. 5). This observation underscores the complexity of pinning down a specific number of “major candidate genes” conditioning the spinach-WR pathogen interaction; for instance, in a recent study using members of the same population, we showed that the width of some LD blocks extended up to 100 Mb^14^, suggesting that large chunks of the genome in this material is are still well conserved and may contain long swathes of with many SNPs intervening in WR disease.

### Variable correlation between markers in the allelic tests and those identified in the GWA analysis

The statistical methods that have been developed to control for population structure may produce a more uniform *P* value distribution but can have reduced sensitivity^13,24,62,63^. The use of the Bonferroni correction (BC) in assigning significance may be highly conservative, however, generating many false negatives^64^ and causing a lower correlation percentage between the significant markers in the GWA and those containing the associated minor alleles (Table 3; Fig. 4). In fact, while 71% of markers identified in the Q1 vs. Q4 allelic association were also significant in the GWA (Table 3), this number increased to 78% when the less conservative Benjamini–Hochberg’s false-discovery rate (FDR) correction was used instead of the BC. Since the allelic tests analyse the association of the minor alleles with the severity of the WR incidences, a vast proportion of the alleles and/or the associated markers not represented among the significant polymorphisms in the GWA may actually have been significant major alleles^4^. We did not test this; however, the fact that the heavily diseased plants invariably showed a higher MAF strongly suggests that these rare variants are indeed important genomic risk factors for WR in the susceptible accessions.

Compared to the GWA model, the allelic tests may have exposed the presence of alleles reflecting stratification in the samples that could not be captured by permutation alone^4,13^. To bridge this gap, Brachi et al.^8^ suggested that the GWAS could be performed on samples partitioned according to regional isolation because they have reduced allelic heterogeneity which is confounded by the population structure. The authors contended that LD blocks are likely to be longer in the regional subsets, a feature that might decrease the mapping resolution. Our data partitioning were not based on the regional identity of the accessions. The accessions included in each segment showed regional heterogeneity in WR resistance and other previously identified phenotypic features (Supplementary Table ST1; https://npgsweb.ars-grin.gov/gringlobal/search.aspx)^14,65,66^. It was not clear in our study whether the partitioning that concentrated the risk alleles to a 25% subset of the population may also have experienced a low mapping resolution, such that many valid signals in the GWA went undetected in the basic allelic tests.

### Different alleles in the same marker

Comparing the nucleotide identities for each given locus, some markers showed the same allelic pair in the GWA analysis as they did in the other models; for instance, of the 15 markers common to both the GWA and in the MS-5, a total of nine SNPs maintained the same minor and major allele sets (see Table 4). Six markers had showed either a complete allele change in the two bases, or just shifts in the major and minor allele frequencies. For example, the markers *18906_141* (chr1, position 31,305,392) and *41441_41* (chr6, 16,966,214) showed T/C and A/T changes, respectively, in both the GWA and in the MS-5 group. On the other hand, the markers *18167_81* (chr1, position 21,843,464) and *23256_116* (chr2, position 40,008,560) showed A/G and G/A polymorphisms, respectively, in the GWA, but were, T/C and C/A polymorphisms, respectively, in the MS-5 (Table 4). These allelic shifts suggest that there may have been be multiple alternate alleles at the same loci^4^, even though the algorithms we used were based on a di-allelic architecture. Multiple alleles in many LD haplotype blocks^67^ may also mask the penetrance of each other^4,68^. Indeed, in a study of berry numbers in a grapevine (*Vitis vinifera*), Myles et al.^13^ identified a number of association signals of for alleles in strong LD regions with containing non-genotyped but functional alleles. This discrepancy in allele identity in this study may also have been an artefact of our artificial in silico “selection pressure” resulting in allelic drift^3^ in favour of the WR-DSR distribution tails. The alleles that are similar in both the MS-5 and the GWA analyses indicate they are common disease variants present in the whole entire population.

### Discrepancies in the heritability estimates

The variations explained by the field phenotyping highly overestimated the heritability (98%) compared to the heritability (64%) determined from the variance components. Similarly, the in the GWA model, the marker based mean heritability was 98% compared with a paltry <10% variation for the most heritable marker. We did not determine marker-based heritability for the markers identified using the allelic association and the GAF models. In any case, these observations underpin the conundrum associated with the “missing heritability” in the GWA models. For the purpose of this study, we will not try to untangle this ambiguity. Better and more extensive coverage of the probable causes of such wide disagreements between the predicted and observed heritability can be found in other studies and reviews^4,8,68^.

### Risk alleles and functional assignment

It has been suggested that many rare variants may be functionless, yet they can still accumulate in the genome and eventually become functional if combined with other nearby variants^4,8^. Such variants may, for example, accumulate over time, and eventually forming novel transcription start sites, transcription factor-binding sites, protein-binding sites, and histone-binding sites^7,69^, which may be functional in pathogen–host plant interactions. In fact, some of these studied functionalities were represented in a small sample of 15 SNPs identified in plants displaying the highest and lowest WR-DSR scores in this study (Fig. 4; Table 4). For instance, among the markers, *18906_141* (chr1, position 31,305,392) is anchored on the gene *Spo00225*, which contains a predominantly plant-based protein domain known as a pentatricopeptide repeat (PPR). PPR mediates many plant functions, including the regulation of gene expression, by binding RNA and negatively regulating abscisic acid signalling and many other plant development and stress signalling pathways^70–72^. The marker *23256_116* (chr2, position 40,008,560) is hosted by the gene *Spo14439*, which encodes Histone H3, the acetylation of which mediates many fungal pathogen–host plant interactions (see Jeon et al. review^73^).

Many of the mapped markers involved in other processes are listed in the Supplementary Tables ST3, ST4, ST5, ST6, and ST7; however, we emphasize that the functionality of these markers in spinach-WR interactions should be understood in the context of genomic regions with extended LD clusters of high-scoring SNPs, which poses a challenge for the selection of WR disease resistance using candidate genes^74^. In addition, the marker spectrum of all the models used in this study (Fig. 5) may underscore the complex pattern of allelic heterogeneity in the WR–plant interactions. In our view, therefore, the most promising genetic control of WR may be through genomic selection.

### Significance for molecular breeding

We have shown that, by segmenting data based on phenotypic strength, the target alleles in individuals with stronger WR phenotypes have a significantly higher MAF than in those showing weaker WR phenotypes. In fact, a marker–phenotype relationship test using the 15 markers in 20 sequences (10 of resistant plants and 10 for susceptible plants) show a discrimination of the resistant group from the susceptible group of accessions (Fig. 6). The striking preponderance to discriminate between the resistance lines from the susceptible lines among the 20 accessions at the corresponding 5th percentiles strongly suggest that 11 markers are resistance factors in the nine MR accessions, while the four markers are susceptibility factors in the 10 MS accessions. Since none of the nine lines have been previously reported as possible resistance sources for white rust of spinach, they may form part of potentially new sources for breeding WR-resistant spinach. The application of this method could narrow down the targeted population to enable a detailed study of the disease resistance associated with these minor alleles. This may be useful in assigning marker significance directly to the important phenotypic tails and for quickly assessing the underlying molecular causative variants and thus provides an insight into the loci required for susceptibility. Furthermore, by focusing on the distribution tails, allelic mapping can potentially be used to identify markers associated with quantitative traits in plant systems while significantly reducing the population size required for testing.

## Supplementary information

Supplementary Table ST1 Phenotypic data and accession information

Supplementary Table ST2 Clustering of phenotypic data

Supplementary Table ST3 Markers and associated significant alleles in the most resistant Q1 and in the most susceptible Q4 accessions

Supplementary Table ST4 Markers and associated significant allelic tests in the resistant Q2 and in the susceptible Q3 accessions

Supplementary Table ST5 Markers and associated significant allelic tests in the most resistant 5% (MR-5) and the most susceptible 5% (MS-5) accessions

Supplementary Table ST6 Markers associated with white rust in the basic genotypic full model association test

Supplementary Table ST7 Significant signals (Bonferroni correction) and related statistics in the genome-wide association analysis
